# Efficiency improvement in the cantilever photothermal excitation method using a photothermal conversion layer

**DOI:** 10.3762/bjnano.7.36

**Published:** 2016-03-10

**Authors:** Natsumi Inada, Hitoshi Asakawa, Taiki Kobayashi, Takeshi Fukuma

**Affiliations:** 1 Division of Electrical Engineering and Computer Science, Kanazawa University, Kanazawa, Japan; 2 Bio-AFM Frontier Research Center, Kanazawa University, Kanazawa, Japan; 3 PRESTO, JST, Kawaguchi, Japan; 4 ACT-C, JST, Kawaguchi, Japan

**Keywords:** atomic force microscopy, cantilever excitation, dynamic mode, photothermal conversion, photothermal excitation

## Abstract

Photothermal excitation is a cantilever excitation method that enables stable and accurate operation for dynamic-mode AFM measurements. However, the low excitation efficiency of the method has often limited its application in practical studies. In this study, we propose a method for improving the photothermal excitation efficiency by coating cantilever backside surface near its fixed end with colloidal graphite as a photothermal conversion (PTC) layer. The excitation efficiency for a standard cantilever of PPP-NCHAuD with a spring constant of ≈40 N/m and a relatively stiff cantilever of AC55 with a spring constant of ≈140 N/m were improved by 6.1 times and 2.5 times, respectively, by coating with a PTC layer. We experimentally demonstrate high stability of the PTC layer in liquid by AFM imaging of a mica surface with atomic resolution in phosphate buffer saline solution for more than 2 h without any indication of possible contamination from the coating. The proposed method, using a PTC layer made of colloidal graphite, greatly enhances photothermal excitation efficiency even for a relatively stiff cantilever in liquid.

## Introduction

Atomic force microscopy (AFM) [1] is an analytical technique to investigate nanoscale surface structures and local physical properties of various samples. Dynamic-mode AFM has attracted considerable interests in various fields due to its great potential for many applications. For example, recent advancements in instrumentation of dynamic-mode AFM have enabled atomic-resolution imaging not only in vacuum [2–4] but also in liquid [5–6]. In addition, other advanced AFM techniques such as high-speed AFM [7–9] and multifrequency AFM [10–12] have been developed based on dynamic-mode AFM. In dynamic-mode AFM, a stiff cantilever is mechanically oscillated at a frequency near its resonance frequency. The vibrational characteristics, such as frequency, amplitude and phase are monitored to detect interaction forces between a sharp tip and a sample. Therefore, an excitation method of cantilever oscillations is an important technique in dynamic-mode AFM.

Acoustic excitation is the most widely used method for cantilever excitation in dynamic-mode AFM. The method is used in many commercially available AFM systems because of its simple setup and high usability. In the method, a cantilever oscillation is excited by vibrating a piezoelectric actuator integrated in a cantilever holder. However, spurious resonances in the surrounding liquid and mechanical parts often deteriorate the stability and accuracy of AFM measurements [13–14]. To solve these problems, alternative methods have been developed such as photothermal excitation [15–17], magnetic excitation [18–19] and electrostatic excitation [20]. In the photothermal excitation method, a power-modulated laser beam irradiates the fixed end of a cantilever. The cantilever oscillation is excited by thermal stress induced by the irradiated laser beam [21]. Owing to the direct excitation of the cantilever, excitation of the spurious resonances is negligible [22].

However, the photothermal excitation method has the disadvantage of low excitation efficiency. Due to the low excitation efficiency, the cantilever oscillation with a desired vibrational amplitude is often difficult to achieve with a moderate laser power (on the order of milliwatts). In particular, a cantilever with a large spring constant requires a large laser power modulation. To overcome this disadvantage, cantilevers are typically coated with a thin metal layer to provide large amplitude response [21,23–25]. The difference in the thermal expansion coefficients between the cantilever material (e.g., silicon or silicon nitride) and thin metal layer (e.g., gold or aluminum) induces a large mechanical stress. Although the metal-coated cantilevers are used in most of the experiments, the excitation efficiency is often insufficient. Therefore, several methods have been proposed to improve the efficiency of the photothermal excitation method. For example, Kiracofe et al. reported that a cantilever with a trapezoidal-shaped cross section showed a higher photothermal efficiency than that with a rectangular-shaped cross section due to difference in thermal distribution in the cantilever [26]. The results indicated that the efficiency of photothermal excitation can be improved by optimizing the cantilever geometry.

As an alternative approach, the improvement of excitation efficiency using a short wavelength laser beam has been reported [27]. The high efficiency when using a short wavelength laser beam compared to a long wavelength laser beam is explained by the optical absorption characteristics of the cantilever material (e.g., silicon was used in [27]). However, the short wavelength light may cause sample damage when biological molecules or organic molecules are studied. To avoid this, an excitation laser with a longer wavelength (e.g., infrared light) is preferred in some cases. Although sample damage can be suppressed by the use of a long wavelength laser beam, the efficiency of photothermal excitation is not as high as that obtained by a short wavelength laser beam. For these reasons, improvement in the photothermal excitation efficiency when using a laser beam with a long wavelength is strongly demanded. Ratcliff et al. reported that a coating layer of black paint or Au/Pd on the cantilever backside enhances the photothermal excitation efficiency by increasing the absorption of the laser light [21]. In this previous study, relatively soft cantilevers with spring constants of 0.58 and 0.12 N/m and a visible laser beam were used. However, since the excitation efficiency decreases with increasing cantilever stiffness (or with increasing the excitation laser beam wavelength), it is important to experimentally confirm the applicability of such a coating method with a relatively stiff cantilever and an infrared excitation laser beam.

In this study, we aimed to improve the photothermal excitation efficiency with relatively stiff cantilevers using a photothermal conversion (PTC) layer made of colloidal graphite. We have established a procedure with a micromanipulator and glass probes to form a PTC layer only at the fixed end of the cantilever to avoid reducing the detection sensitivity of the optical beam cantilever deflection sensor. We demonstrate improvement in cantilever excitation efficiency by using a PTC layer with two types of commercially available cantilevers with nominal spring constants of 42 and 85 N/m (PPP-NCHAuD and AC55). In addition, we demonstrate high stability of the PTC layer in liquid by long-term FM-AFM imaging of mica with atomic resolution in phosphate buffer saline (PBS) solution.

## Results and Discussion

### Preparation of PTC layers

**Figure 1:**
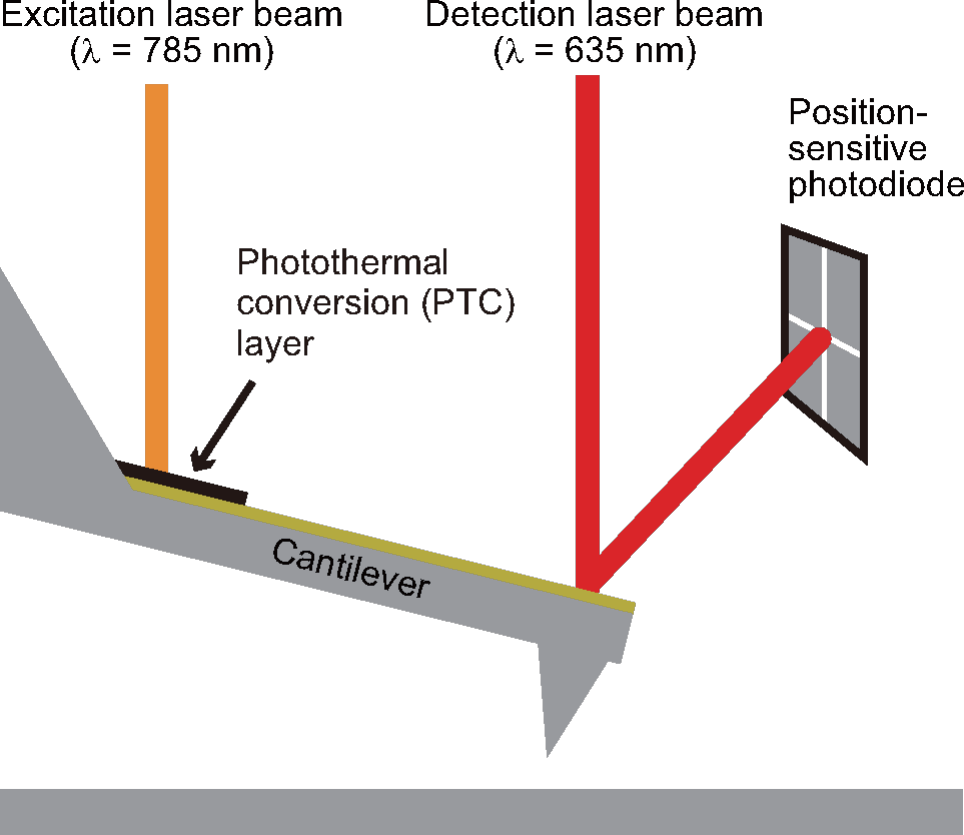
Schematic illustration of the photothermal excitation setup using a cantilever coated with a PTC layer.

Figure 1 shows a dynamic-mode AFM setup with two laser beam sources for detection of cantilever deflection and photothermal excitation. The detection and excitation laser beams are irradiated onto the free end and fixed end of a cantilever, respectively. In this study, we chose colloidal graphite as the PTC layer material. This is because carbon materials (e.g., graphite and CNT) provide a high efficiency in conversion of light to heat [28–30] and hence are used in various fields such as printing technology and thermal-type infrared sensing. Since colloidal graphite shows a high absorption efficiency at wide wavelength range [31–32], it may be used for improving the photothermal excitation efficiency. Meanwhile, the cantilever free end should not be coated with a PTC layer because the detection laser beam is irradiated at this position. Thus, a method for coating only at a small region near the cantilever fixed end is necessary. We have established a coating method for a PTC layer of colloidal graphite using a micromanipulator (AxisProSS, Microsupport, Shizuoka, Japan). In this study, we tested PTC layers on two types of commercially available cantilevers: (1) PPP-NCHAuD (Nanoworld, Neucatel, Switzerland) is widely used for dynamic-mode AFM measurements in liquid and (2) AC55 (Olympus, Tokyo, Japan) is a relatively stiff cantilever with a smaller size than that of PPP-NCHAuD. The backsides of both cantilevers were coated with a thin gold layer.

**Figure 2:**
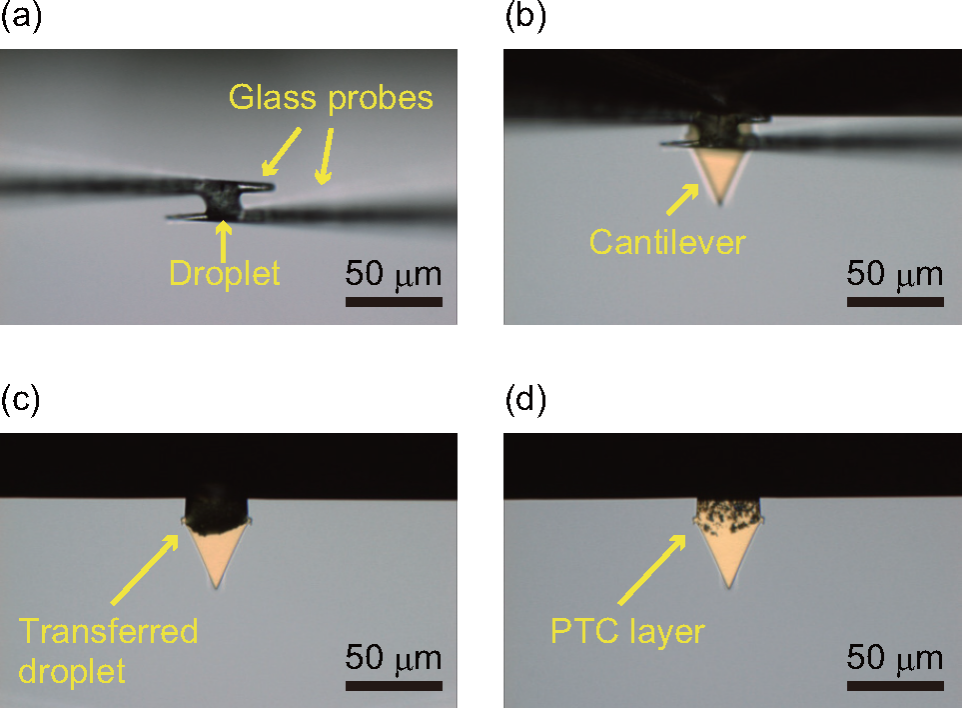
Formation of a PTC layer at a cantilever fixed end with a micromanipulator. (a) Preparation of a small droplet with a diameter of 20 μm by glass probes. (b) Small droplet is deposited on the cantilever fixed end. (c) Before drying. (d) After drying.

Figure 2 shows the coating process of a PTC layer on an AC55 cantilever. A small droplet of colloidal graphite dispersion was formed using two glass probes that were controlled by the micromanipulator. The diameter of the small droplet was approximately 20 μm. We found that the coating with aqueous solution was difficult due to water evaporation. Thus, glycerol (23 wt % of total liquid weight) was added to the coating solution. The addition of glycerol enables highly reproducible coating of the PTC layer. To remove the glycerol and water, the coated cantilever was heated at 200 °C for 2 h under reduced pressure (*<*3 × 10^−3^ Pa) using a vacuum oven.

**Figure 3:**
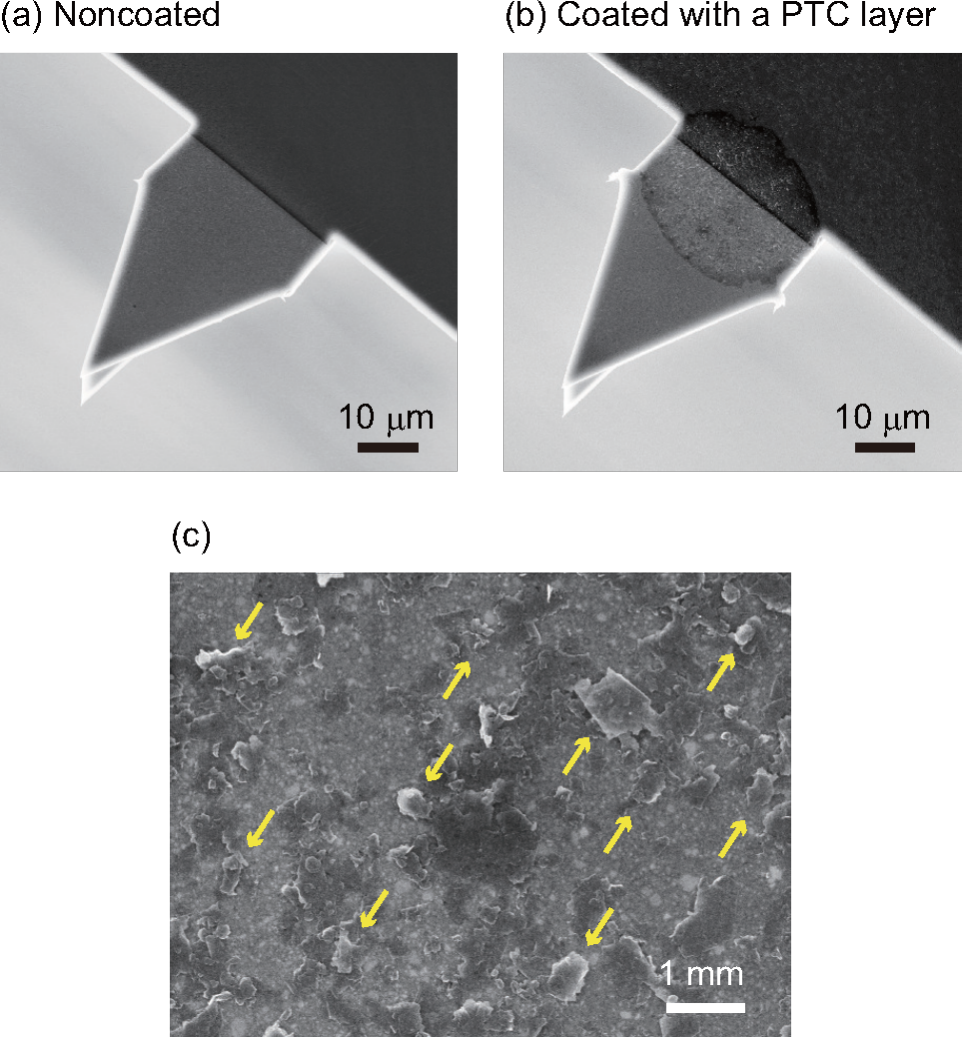
SEM images of AC55 cantilevers. (a) Noncoated and (b) coated with a PTC layer. (c) A magnified SEM image of the PTC layer. The arrows indicate plate-like colloidal graphite.

Figure 3a,b shows SEM images of PTC layers on AC55 cantilevers before and after the coating. The results suggest that a PTC layer was formed only at a small region near the cantilever fixed end. Thus, the PTC layer should give little influence on the cantilever deflection measurements. In the magnified SEM image (Figure 3c), we found plate-like particles with a diameter between 0.1 and 1 μm. The diameters observed in the SEM images agree with the average diameter of the colloidal graphite (460 nm) measured by dynamic light scattering. The results show that the plate-like particles observed in the SEM images are colloidal graphite.

### Performance of PTC layers

**Figure 4:**
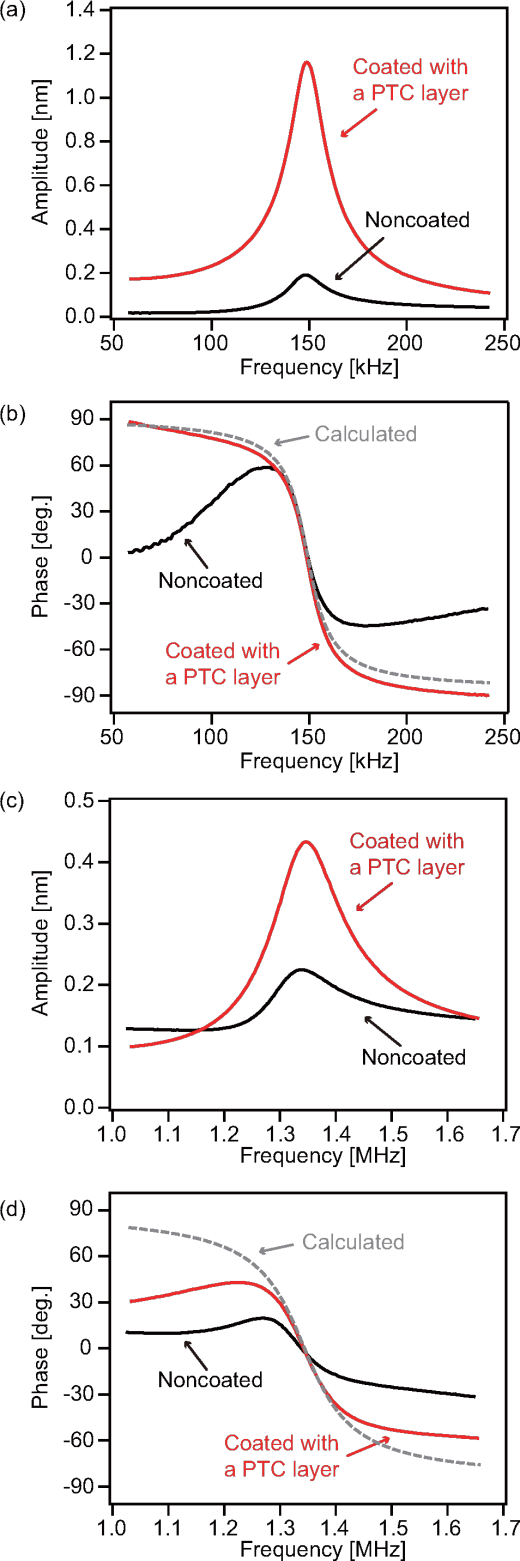
(a) Amplitude and (b) phase versus frequency curves measured with a PPP-NCHAuD in water. (c) Amplitude and (d) phase versus frequency curves measured with an AC55 cantilever in water (Cantilever (iii) in Figure 5a). All curves were measured with the same amplitude of laser power modulation (*P*_mod_ = 12.9 mW). The dimensions of cantilevers are significant different between PPP-NCHAuD (length; 125 μm, width; 30 μm, thickness; 4 μm) and AC55 (length; 55 μm, width; 31 μm, thickness; 2 μm).

Figure 4 shows amplitude and phase versus frequency curves measured with two different types of cantilevers (PPP-NCHAuD and AC55) before and after coating of the PTC layer. To evaluate performance of the PTC layers, we measured the sweep curves with photothermal excitation in water. The amplitude curves obtained for the PPP-NCHAuD cantilever (Figure 4a) show that the peak amplitude measured with the coated cantilever is six times higher than that with the noncoated cantilever. The results suggest the effectiveness of a PTC layer for improving the photothermal excitation efficiency. For a relatively stiff AC55 cantilever, the increase of the peak amplitude is approximately two times. This improvement is not as high as that obtained for a softer PPP-NCHAuD cantilever. However, a doubled increase of the excitation efficiency has significant merit for use of relatively stiff cantilevers in many practical applications as they are difficult to oscillate with a sufficient amplitude.

The lower increase rate of the stiff AC55 cantilever compared to the soft PPP-NCHAuD cantilever is likely to be caused by multiple reasons. However, a quantitative comparison of the increase rates between two cantilevers is difficult in this study due to the use of different objective lenses. Thus, we discuss possible reasons for the large difference in the increase rates. The most likely reason is the difference in the three-dimensional shape of the cantilevers. The two cantilevers have different cross-sectional shapes: AC55 cantilever has a rectangular cross section, and PPP-NCHAuD has a trapezoidal cross section. In addition, they have a large difference in the dimensions (length, width and thickness) as shown in the caption of Figure 4. The excitation efficiency and optimal irradiation positions of an excitation laser should be affected by the three-dimensional shapes of cantilevers as previously reported in [26].

The phase versus frequency curves (Figure 4b,d) show the improvement of the phase response by PTC layer coating. The phase curves measured with the noncoated and coated cantilevers were corrected by subtracting the frequency-dependent phase delay caused by a phase-locked loop circuit. The dotted lines in the figures show ideal phase curves calculated with resonance frequency (*f*_0_) and *Q*-factor estimated from cantilever thermal vibration spectra as shown in Table 1 and Table 2. The phase curves measured with noncoated cantilevers were not consistent with the calculated ideal curves. In contrast, the curve measured with a coated PPP-NCHAuD showed almost the same profile as that of the ideal one (Figure 4b). In addition, the curve measured with a coated AC55 showed the improvement of phase response compared to that measured with the noncoated AC55 cantilever (Figure 4d). The errors in the measured curves were mostly caused by a reflection of the excitation laser beam into the photodetector and the low excitation efficiency. The results suggest that the coating of a PTC layer improves the phase response obtained by the photothermal excitation method.

**Table 1:** Properties of a PPP-NCHAuD cantilever before and after coating with a PCT layer.

	*f*_0_ [kHz]	*Q*	*k* [N/m]	*A* [nm]	η_exp_ [nm/mW]	Blackened area [%]

Noncoated	149	8.5	35.6	0.19	0.015	—
Coated	149	8.5	36.3	1.16	0.090	100

**Table 2:** Properties of AC55 cantilevers before and after coating with PCT layers.

	*f*_0_ [MHz]	*Q*	*k* [N/m]	*A* [nm]	η_exp_ [nm/mW]	Blackened area [%]

Noncoated (ii)	1.28	12.9	107	0.25	0.020	—
Coated (ii)	1.28	11.0	121	0.38	0.030	35
Noncoated (iii)	1.34	12.0	132	0.22	0.018	—
Coated (iii)	1.34	10.2	142	0.43	0.034	55
Noncoated (iv)	1.35	10.5	101	0.18	0.014	—
Coated (iv)	1.35	10.7	141	0.44	0.035	70
Noncoated (v)	1.36	11.0	133	0.20	0.016	—
Coated (v)	1.38	10.4	129	0.30	0.024	97

Table 1 and Table 2 show the physical properties of PPP-NCHAuD and AC55 cantilevers before and after coating with a PTC layer. The resonance frequency (*f*_0_), *Q*-factor and spring constant (*k*) of the cantilevers were estimated from cantilever thermal vibration spectra obtained in water. We found that the PTC layers coating had little influence on the physical properties of these two types of cantilevers. Thus, a PTC layer should not change cantilever performance, such as force sensitivity.

### Relationship between excitation efficiency and blackened area with PTC layers

The increase rate in excitation efficiency of a PPP-NCHAuD cantilever (six times) was sufficient for most of the practical applications of dynamic-mode AFM in liquid. In addition, the phase response was also improved and was very close to the ideal curve as shown in Figure 4b. In contrast, the improvements in the excitation efficiency and the phase response obtained with the stiff AC55 cantilever were lower than those obtained with the soft PPP-NCHAuD cantilever. Therefore, we investigated a relationship between excitation efficiency and blackened area with PTC layers on AC55 cantilevers for further improvements.

We found that the photothermal excitation efficiency of the coated cantilevers shows large variation depending on the coating conditions of a PTC layer. Initially, we tried to optimize the excitation efficiency by reducing the graphite concentration in the dispersions. Coarse regulation of the excitation efficiency was possible by this method. However, fine regulation only by controlling the graphite concentration was difficult due to the difference in drop volumes formed by two glass probes and the inhomogeneity of colloidal graphite flakes in the dispersions. Owing to these reasons, it is difficult to estimate the accurate excitation efficiency only from the graphite concentration. To solve this problem, we found the relationship between the blackened area evaluated by optical microscopy and excitation efficiency.

**Figure 5:**
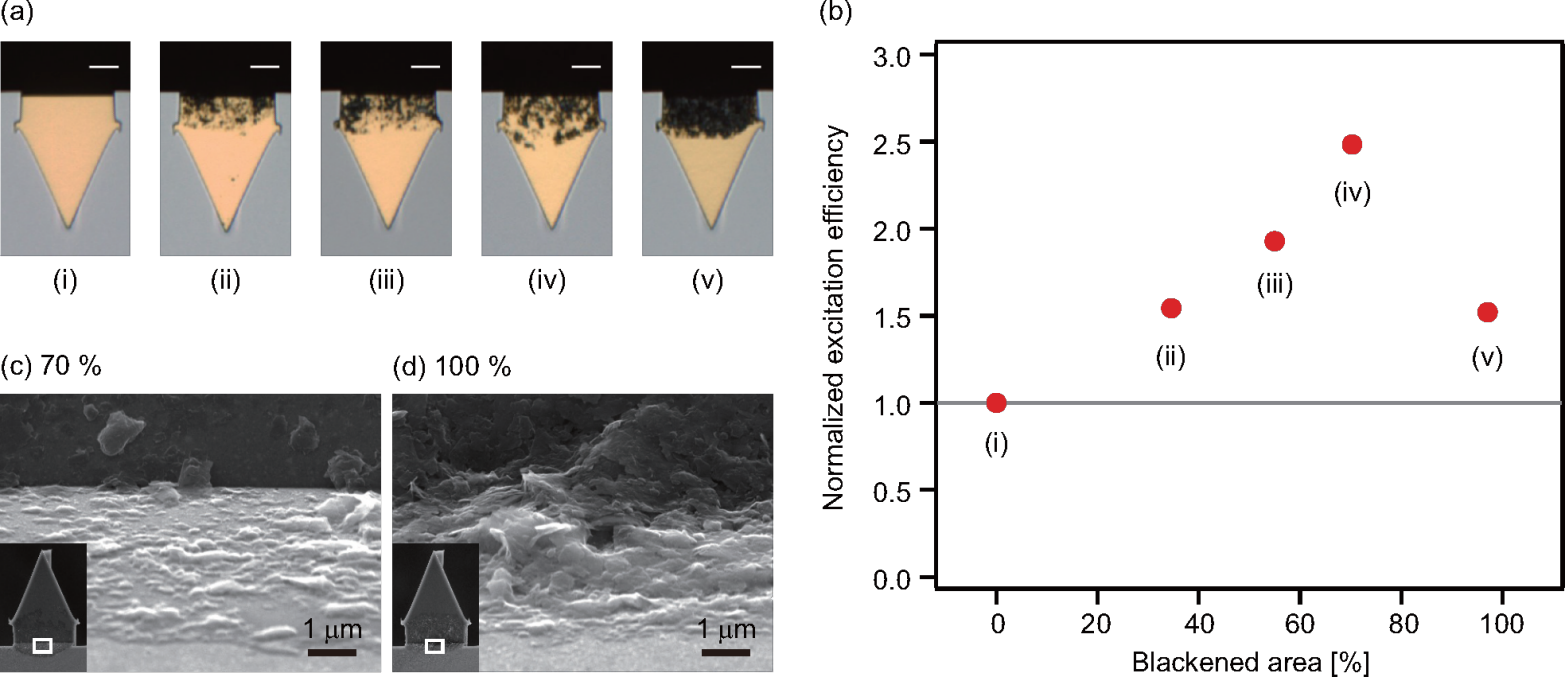
(a) Optical images of AC55 cantilevers having different surface coverage. (i): Noncoated cantilever. The cantilevers were coated with the dispersions in the colloidal graphite concentration of (ii) 0.4 wt %, (iii) 4 wt %, (iv) 2 wt % and (v) 6 wt %. (b) Dependence of excitation efficiency on blackened area of a PTC layer. AC55 cantilevers were used in this experiment. (c, d) SEM images of cantilevers with 70 and 100% blackened area. The rectangles in the insets indicate the location where we took a magnified image.

We coated cantilevers with different blackened areas as shown in Figure 5a. The blackened areas near the cantilever fixed end were calculated by a method described in the experimental section. Amplitude and phase versus frequency curves measured with the cantilevers in Figure 5a are shown in Supporting Information File 1, Figure S1. The results suggest that the peak values of the amplitude versus frequency curves measured with these cantilevers are all increased by the coating of the PTC layers. In addition, the phase responses are improved with increasing blackened area. Figure 5b shows the blackened area dependence of the photothermal excitation efficiency (η_exc_). Here, we define η_exc_ as


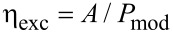


where *A* and *P*_mod_ are the peak value of an amplitude versus frequency curve and the modulation amplitude of the excitation laser power, respectively. The result shows that η_exc_ increases with blackened area coverage up to about 70%. This is probably due to the improvement in the photothermal conversion efficiency.

In contrast, the photothermal excitation efficiency remarkably decreases with increasing blackened area from 70 to 100%. To understand the reason for the decrease, we imaged the cantilevers with blackened area of 70 and 100% by SEM (Figure 5c,d). The SEM images show that the PTC layer with 100% blackened area is much thicker than the one with 70% blackened area. In addition, the PTC layer with 100% blackened area shows relatively large roughness compared with the one with 70%. The large roughness of 100% blackened area in the SEM image indicates that the flakes of colloidal graphite are likely to stack on the surface of the cantilever with hollow spaces. The hollow spaces in the PTC layer may cause the decrease in heat transfer from the PTC layer to the cantilever.

Another possible mechanism is an influence of heat transfer in the lateral direction by connected flakes of colloidal graphite. The lateral connection of colloidal graphite may lead to the increase of heat transfer in the lateral direction, resulting in a small thermal gradient in the cantilever. In fact, the SEM image of the 100% blackened area (Figure 5d) shows that the colloidal graphite flakes are connected. In contrast, most of the flakes are isolated and directly attached to the surface of the cantilever in the 70% blackened SEM image (Figure 5c). The results support that the generated heat is efficiently transmitted to the cantilever with low heat transfer in the lateral direction, resulting in an increase of generated mechanical stress.

The results indicate that the optimal coating of the PTC layer may be slightly lower than 100% as long as a multilayered structure with hollow spaces and/or a lateral connections between the flakes of colloidal graphite are not formed. However, reproducible formation of such a PTC layer is difficult with the present coating method using the micromanipulator and glass probes. Since even a slight increase from 70% blackened area results in a remarkable decrease in the excitation efficiency, we used PTC layers with a blackened area of ≈70% in our experiments.

### Long-term stability of PTC layers in liquid

**Figure 6:**
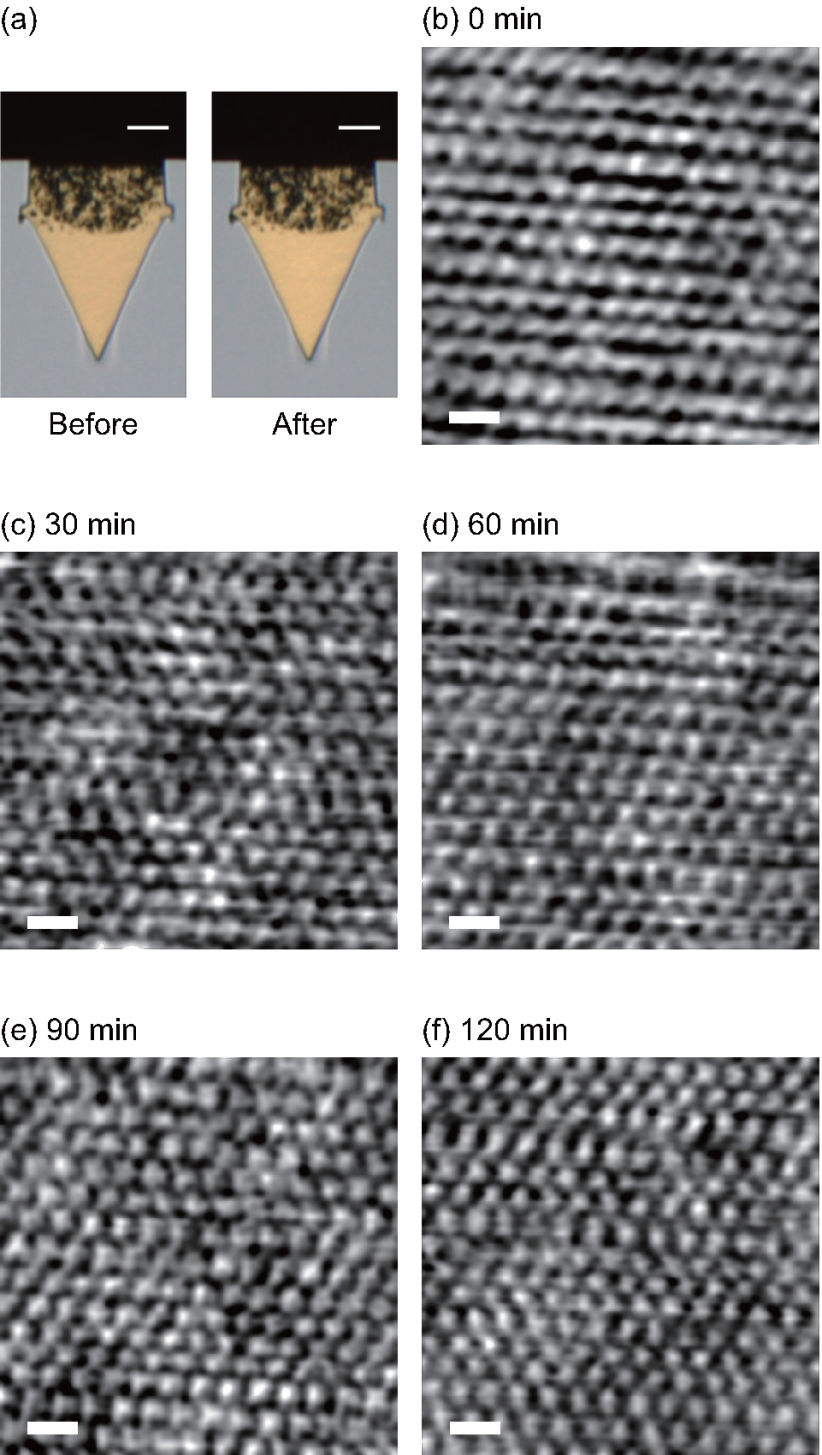
Long-term stability of the PTC layer in liquid. (a) Optical images of an AC55 cantilever before and after use in water. (b)–(f) Successive FM-AFM images of a mica surface in PBS solution. Δ*f* = +3.9 kHz. *A* = 0.4 nm. Scale bar = 1 nm.

Long-term stability of a PTC layer in liquid is very important for stable operation of a photothermal excitation system in dynamic-mode AFM. To investigate the long-term stability, we measured η_exc_ for 2 h in water with an AC55 cantilever coated with a PTC layer (as shown in Supporting Information File 1, Figure S2). The result reveals that the photothermal excitation efficiency is extremely stable in water. Furthermore, we confirmed that the optical microscope images of the PTC layer before and after the measurement are almost the same (Figure 6a).

Figure 6b–f shows FM-AFM images of a mica surface obtained in PBS solution using an AC55 cantilever coated with a PTC layer. After adjusting the imaging parameters such as Δ*f*, *A* and feedback gains to obtain atomic resolution, long-term FM-AFM imaging was performed for 2 h without changing the imaging parameters. We found subnanometer-scale contrasts corresponding to the mica surface structure in all the successive AFM images. In addition, no contaminations on the mica surface were found in the AFM images. The results show that a PTC layer does not have any negative influence on the atomic-scale FM-AFM imaging in liquid.

## Conclusion

In this study, we proposed a method for improving the photothermal excitation efficiency in dynamic-mode AFM using a PTC layer made of colloidal graphite. We have established a procedure to prepare a PTC layer only at the cantilever fixed end. The photothermal excitation efficiency increases with increasing blackened area of colloidal graphite up to about 70%. In contrast, the excitation efficiency remarkably decreases with increasing the blackened area from 70 to 100%. The results indicate that the decrease is due to formation of multilayered structures of colloidal graphite with hollow spaces and/or lateral connections between flakes. A PTC layer provides six-fold improvement in the excitation efficiency for a standard PPP-NCHAuD cantilever while over two-fold for a stiffer AC55 cantilever. Such an improvement is particularly useful for oscillating a relatively stiff cantilever with a long wavelength laser beam. We experimentally demonstrated the high stability of PTC layers in liquid by the long-term measurements in water and PBS solution. The proposed method should extend the applicability of the photothermal excitation method.

## Experimental

### Preparation of coating solution used for the formation of PTC layers

A commercially available aqueous dispersion of colloidal graphite (graphite 5–10% and ammonium hydroxide 1–5% in water, Aquadag E, Henkel, Düsseldorf, Germany) was used as the PTC layers material. To control the surface coverage of colloidal graphite on a cantilever, the aqueous dispersion was diluted with Milli-Q water. We added glycerol (Nacalai Tesque, Kyoto, Japan) to each aqueous dispersion to obtain a final concentration of 23 wt % in order to prevent water evaporation in the coating process. The colloidal graphite concentration of the dispersions used in this study are shown in the caption of Figure 5. Sonication of the coating solution was performed before the coating process. The average diameter of colloidal particles in the coating solution was measured by the dynamic light scattering (Zetasizer Nano-ZS, Malvern, Worcestershire, UK).

### Measurement of photothermal excitation efficiency

A custom-built AFM equipped with a photothermal excitation setup and a commercially available oscillation controller (Nanonis OC4, SPECS, Zürich, Switzerland) were used for the photothermal excitation efficiency measurement. An infrared laser (λ = 785 nm, Melles Griot, Irvine, CA, USA) was used as an excitation laser source as shown in Figure 1. The laser power was modulated with an external voltage signal from the oscillation controller. The power-modulated laser light was focused on a cantilever fixed end through a collimator lens (F220FC-780, Thorlabs, Newton, USA) and an objective lens (CF Plan Epi 5× for PPP-NCHAuD and CF Plan Epi 10× for AC55, Nikon, Tokyo, Japan). The laser power was measured just after passing through the optical lenses by an optical power meter. The position of laser spot was adjusted near the cantilever fixed end to maximize the amplitude of cantilever oscillation.

### Optical and SEM imaging of PTC layers

The PTC layers were imaged by an optical microscope integrated in the micromanipulator system. To calculate the blackened area of the PTC layers near the cantilever fixed end, the optical images were taken under the same illumination condition. The obtained optical images were processed using an image processing software (ImageJ [33]). The small areas (10 μm × 10 μm) near the cantilever fixed ends were cut out from optical images and converted to 8-bit gray scale images. The regions coated with colloidal graphite in the gray scale images were selected using a function of Make Binary in ImageJ software. The threshold value of 110 was manually chosen to separate the coated and noncoated regions. SEM (JSM-7100F, JEOL, Tokyo, Japan) was used for imaging colloidal graphite on the cantilevers.

### Long-term stability evaluation of PTC layers in liquid

Long-term stability of the PTC layers in liquid was evaluated by monitoring the cantilever excitation amplitude. The signal of excitation amplitude from the oscillation controller (OC4) was recorded by a data logger (ZR-RX40, Omron, Tokyo, Japan) for 2 h every 10 s. An AFM tip was placed far away from the surface (*>*5 mm) to avoid possible influence of tip–sample interactions.

Long-term FM-AFM imaging in liquid was performed using a custom-built AFM with a low-noise cantilever deflection sensor [34–35] and a commercially available phase-locked loop circuit (OC4, SPECS, Zürich, Switzerland). A commercially available AFM controller (ARC2, Asylum Research, Santa Barbara, CA, USA) was used for the tip–sample distance feedback regulation and acquisition of FM-AFM images. The FM-AFM imaging of a mica surface was performed in PBS solution.

## Supporting Information

File 1 Additional figures.
