# Supplementary material for: Efficiency improvement in the cantilever photothermal excitation method using a photothermal conversion layer
Source: Beilstein J Nanotechnol. 2016 Mar 10;7:409–17. doi: 10.3762/bjnano.7.36 (PMC4901543; doi:10.3762/bjnano.7.36)
Supplement: File 1 — Additional figures. [file Beilstein_J_Nanotechnol-07-409-s001.pdf]

Supporting Information

for

Efficiency improvement in the cantilever photothermal excitation method using a photothermal conversion layer

Natsumi Inada[1], Hitoshi Asakawa*[1,2,3], Taiki Kobayashi[1] and Takeshi Fukuma[1,2,4]

Address: [1]Division of Electrical Engineering and Computer Science, Kanazawa University, Kanazawa, Japan; [2]Bio-AFM Frontier Research Center, Kanazawa University, Kanazawa, Japan; [3]PRESTO, JST, Kawaguchi, Japan and [4]ACT-C, JST, Kawaguchi, Japan

Email: Hitoshi Asakawa - hi_asa@staff.kanazawa-u.ac.jp

* Corresponding author

Additional figures

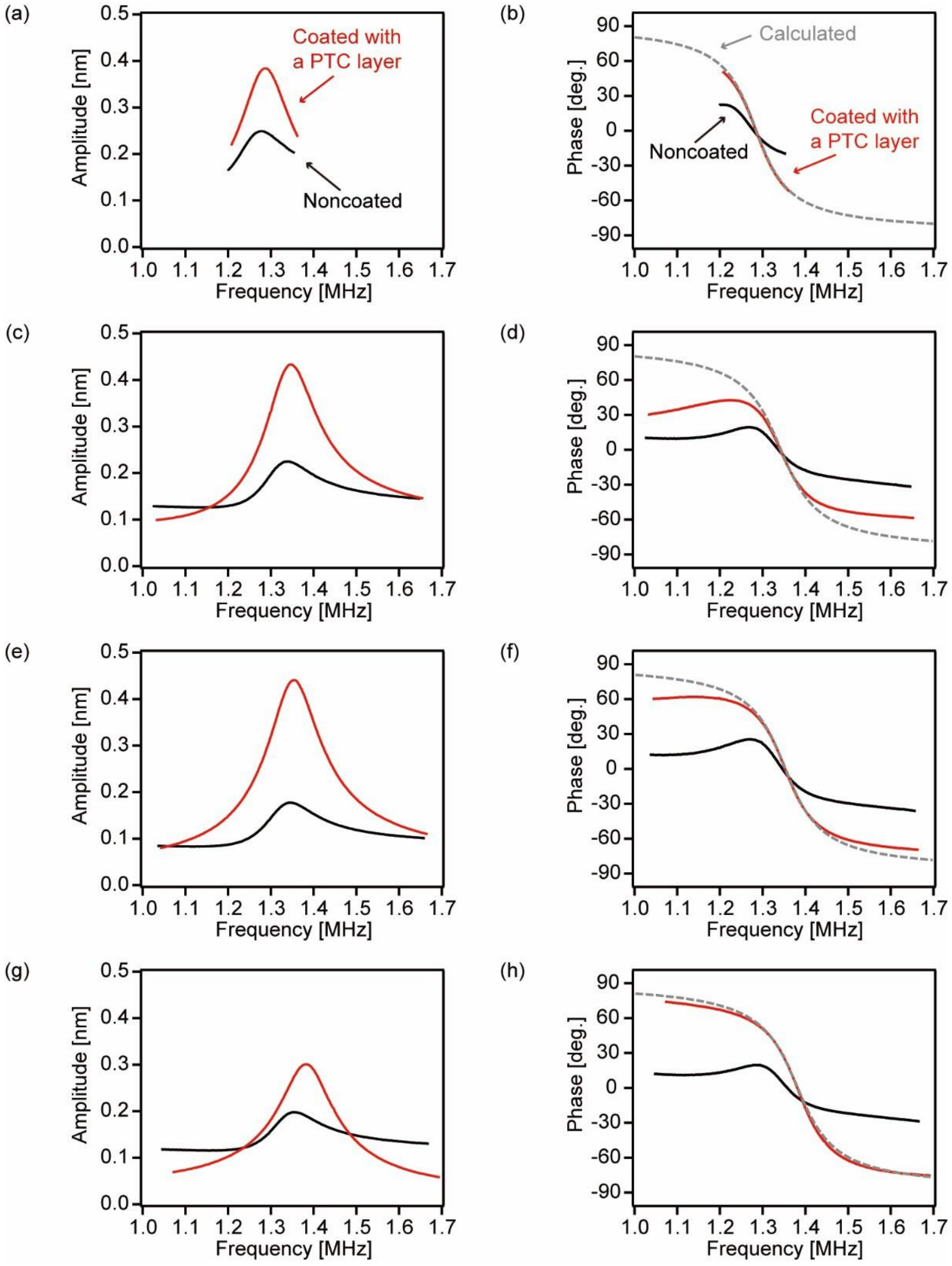

Figure S1: Amplitude and phase versus frequency curves measured with AC55 cantilevers in pure water. (a,b), (c,d), (e,f) and (g,h) were measured with the cantilevers of (ii), (iii), (iv) and (v) in Figures 5a and 5b. All curves were measured with the same amplitude of laser power modulation (P_{mod} = 12.9 mW).

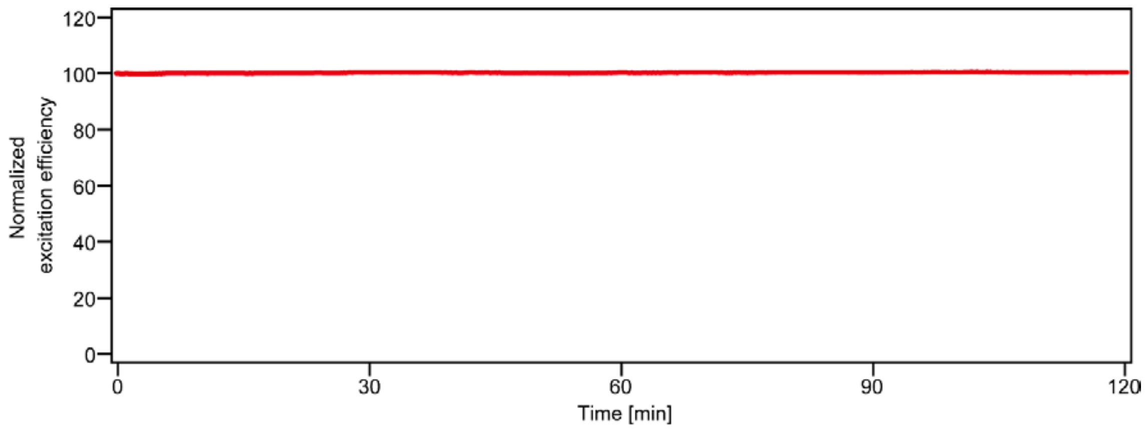

Figure S2: Time dependence of excitation efficiency in water. The oscillation amplitude of the cantilever was kept constant (A = 0.28 nm).
